# Simultaneous Transcriptome Analysis of Sorghum and *Bipolaris sorghicola* by Using RNA-seq in Combination with *De Novo* Transcriptome Assembly

**DOI:** 10.1371/journal.pone.0062460

**Published:** 2013-04-30

**Authors:** Takayuki Yazawa, Hiroyuki Kawahigashi, Takashi Matsumoto, Hiroshi Mizuno

**Affiliations:** 1 Division of Genome and Biodiversity Research, National Institute of Agrobiological Sciences (NIAS), Tsukuba, Ibaraki, Japan; 2 Hitachi Government & Public Corporation System Engineering, Ltd, Koto-ku, Tokyo, Japan; University of the West of England, United Kingdom

## Abstract

The recent development of RNA sequencing (RNA-seq) technology has enabled us to analyze the transcriptomes of plants and their pathogens simultaneously. However, RNA-seq often relies on aligning reads to a reference genome and is thus unsuitable for analyzing most plant pathogens, as their genomes have not been fully sequenced. Here, we analyzed the transcriptomes of *Sorghum bicolor* (L.) Moench and its pathogen *Bipolaris sorghicola* simultaneously by using RNA-seq in combination with *de novo* transcriptome assembly. We sequenced the mixed transcriptome of the disease-resistant sorghum cultivar SIL-05 and *B. sorghicola* in infected leaves in the early stages of infection (12 and 24 h post-inoculation) by using Illumina mRNA-Seq technology. Sorghum gene expression was quantified by aligning reads to the sorghum reference genome. For *B. sorghicola*, reads that could not be aligned to the sorghum reference genome were subjected to *de novo* transcriptome assembly. We identified genes of *B. sorghicola* for growth of this fungus in sorghum, as well as genes in sorghum for the defense response. The genes of *B. sorghicola* included those encoding Woronin body major protein, LysM domain-containing intracellular hyphae protein, transcriptional factors CpcA and HacA, and plant cell-wall degrading enzymes. The sorghum genes included those encoding two receptors of the simple eLRR domain protein family, transcription factors that are putative orthologs of OsWRKY45 and OsWRKY28 in rice, and a class III peroxidase that is a homolog involved in disease resistance in the Poaceae. These defense-related genes were particularly strongly induced among paralogs annotated in the sorghum genome. Thus, in the absence of genome sequences for the pathogen, simultaneous transcriptome analysis of plant and pathogen by using *de novo* assembly was useful for identifying putative key genes in the plant–pathogen interaction.

## Introduction

Plants possess mechanisms to resist pathogen invasion. One of the mechanisms is perception of signals by receptors that act as a surveillance system to recognize pathogens and activate immune responses [1], [2]. Endogenous and exogenous signals provided by MAMPs (microbe-associated molecular pattern molecules), DAMPs (damage-associated molecular pattern molecules), virulence factors, and secreted proteins are recognized directly or indirectly by the receptors. Subsequently, the mitogen-activated protein kinase (MAPK) cascade and Ca^2+^ signaling transmit the signals [3]. Finally, several transcriptional factors (TFs) are activated to regulate the expression of defense genes [1], [4]. This induces several defense responses, such as reinforcement of cell walls, production of phytoalexins, and synthesis of pathogenesis-related (PR) proteins [5]. However, pathogens have evolved mechanisms to attack and penetrate plants. For disease development, pathogens overcome constitutive barriers, avoid or suppress immune responses, and reprogram the host cell so as to establish infectious structures during penetration, invasion, and reproduction [6].

For a comprehensive understanding of plant–pathogen interactions, it is valuable to analyze gene expression in the two interacting organisms simultaneously [7], [8]. However, many studies have physically separated the pathogen and host cells before gene expression analysis. The recently developed RNA sequencing (RNA-seq) technique provides a conceptually novel approach to the study of transcriptomes and would, in principle, allow host and pathogen transcriptomes to be analyzed in parallel. By using this technique, transcriptomes of rice and blast fungus can be analyzed simultaneously [8]. However, RNA-seq often relies on aligning reads to a reference genome and is thus unsuitable for samples from organisms with genomes that are partially or fully unsequenced. *De novo t*ranscriptome assembly provides a way to overcome this limitation. This method assembles RNA-seq reads into transcripts without the aid of a reference genome and in theory allows researchers to reconstruct the sequences of full transcriptomes, identify all the expressed genes, separate isoforms, and capture the expression levels of transcripts [9], [10].

One interesting example of the plant–pathogen interaction is that of *Sorghum bicolor* and *Bipolaris sorghicola*. Sorghum is a C4 grass that is especially important as forage and as a human staple worldwide–especially in the semiarid tropics because of its tolerance of hot and dry environments. *Bipolaris sorghicola* is a necrotrophic fungus that causes a sorghum disease called target leaf spot; the lesions on infected leaves are commonly reddish purple with straw-colored centers. The genomic approach to elucidating the mechanism of this disease started after the sorghum genome was sequenced in 2009 [11]. Previous analyses have focused mainly on positional cloning of a resistance gene [12] or on genes encoding enzymes for secondary metabolism at a relatively late stage of infection [13]. Global transcriptome analysis has revealed that pathogen infection dramatically changes the expression of particular paralogs; these paralogs putatively encode enzymes involved in the synthesis of sorghum-specific phytochemicals that may be responsible for the reddish-purple appearance of infected leaves [13]. Interestingly, sorghum has lines that are resistant to target leaf spot; the lesions in the resistant line SIL-05 are small and chlorotic and clearly differ from the elongated lesions in susceptible lines. Are molecules derived from *B. sorghicola* attacking the immune response of sorghum at an early stage of infection? Because the genome sequence of *B. sorghicola* is not available, the genes of *B. sorghicola* that are expressed during infection are largely unknown. In addition, as sorghum has many proximally duplicated genes [11], the particular genes expressed under the conditions appropriate for specific immune responses have not been fully identified. This strongly limits our understanding of the interaction between sorghum and *B. sorghicola*.

Here, we analyzed the transcriptomes of sorghum and *B. sorghicola* simultaneously by using RNA-seq in combination with *de novo* transcriptome assembly. We used the disease-resistant cultivar SIL-05 and collected samples 12 and 24 h after infection to analyze the early infection stages. Gene expression in sorghum was quantified by aligning reads to a reference genome. For *B. sorghicola*, reads that could not be aligned to the sorghum reference genome were assembled by using the *de novo tra*nscriptome assembler Oases [14]. Several genes that are potentially involved in the growth of *B. sorghicola* on sorghum were expressed in the fungus, and the expression of genes involved in the immune response was induced in sorghum. Thus, we successfully analyzed the transcriptomes of plant and pathogen simultaneously despite limited availability of genomic sequences, and we revealed putative key factors in the plant–pathogen interaction.

## Results

### Overview of Analysis

**Figure 1:**
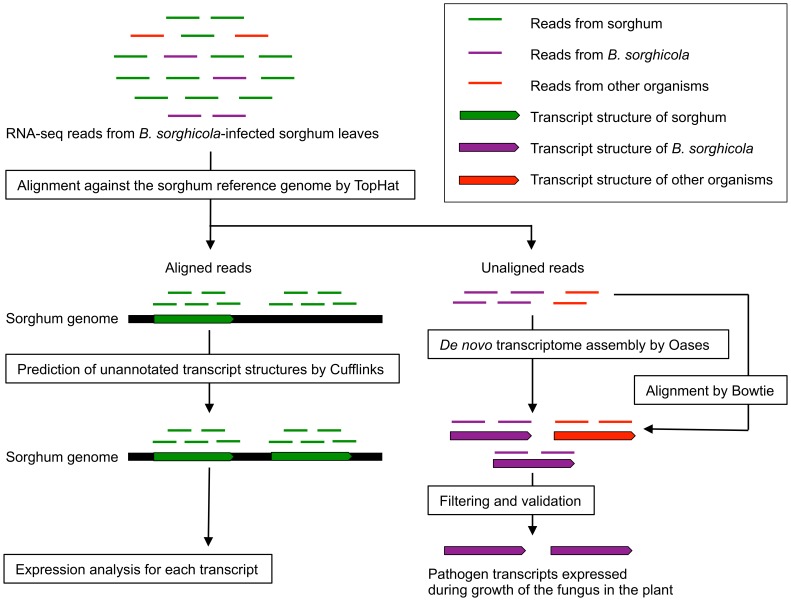
Workflow of analysis. Transcriptomes of sorghum and *Bipolaris sorghicola* were analyzed simultaneously by using different approaches. The mixed transcriptome obtained from *B. sorghicola*-infected sorghum leaves was sequenced by using Illumina mRNA-Seq technology. The sequenced reads contain the reads derived from sorghum (green bars), *B. sorghicola* (purple bars), and other organisms such as normal inhabitants of plant tissue (red bars). The reads were aligned to the sorghum reference genome (black bars), and the aligned and unaligned reads were used to analyze gene expression in sorghum (left) and *B. sorghicola* (right), respectively. Gene expression in sorghum was analyzed for each transcript (green arrows), including transcripts annotated with Phytozome and unannotated transcripts identified on the basis of the piling-up of aligned reads by using the Cufflinks program. For *B. sorghicola*, unaligned reads were assembled by using the Oases program to retrieve the pathogen transcripts expressed during growth of the fungus in the plant. Expression of the assembled transcripts was analyzed by aligning the reads back to the transcripts. The assembled transcripts contained not only the transcripts of *B. sorghicola* (purple arrows) but also those of other organisms (red arrows). The transcripts of other organisms were removed as contaminants, and those of *B. sorghicola* was validated experimentally.

The workflow of the analysis is summarized in Figure 1. We performed mRNA-seq in the target-leaf-spot-resistant cultivar SIL-05 infected with *B. sorghicola*. We collected *B. sorghicola*-infected or mock-infected leaves 12 and 24 h after infection. We then sequenced 100-base-pair single-end reads from mRNAs of the collected leaves by using Illumina mRNA-Seq technology. Of the 31 to 39 million quality-evaluated reads, about 95% were aligned onto the reference genome (Table S1). We analyzed gene expression in *B. sorghicola* by using *de novo* assembly and that in sorghum by using gene models on the sorghum genome (Figure 1).

### Identification of *B. sorghicola* Transcripts by using *de novo* Assembly

For *B. sorghicola* we used a *de novo* transcriptome assembly method, because the whole genome sequence for this organism is not available. A total of 232,760 transcripts were assembled from those reads that could not be aligned onto the sorghum reference genome. The assembled transcripts contained many redundant sequences and may have been contaminated with transcripts from non-*B. sorghicola* organisms, such as normal inhabitants of plant tissue. Therefore, the transcripts were subjected to various filterings to retrieve the non-redundant and reliable pathogen transcripts (see Materials and Methods). To quantify the expression of each transcript, we aligned the reads used for the assembly back to the transcripts and adopted RPKM (Reads Per Kilobase of exon model per Million mapped reads) [15]. For functional annotation of the transcripts, we used the top hit of a BLAST search in RefSeq [16] or the Swiss-Prot database [17].

Finally, we retrieved 160 *B. sorghicola* transcripts that were expressed during growth in sorghum. The transcripts had an average length of 797 bp, a median length of 672 bp, maximum length of 2712 bp, and minimum length of 250 bp. Among these 160 transcripts, we focused on 16 that seemed to be related to growth of *B. sorghicola* in sorghum (Table 1). To evaluate whether the 16 transcripts were of *B. sorghicola* origin, we performed genomic PCR and found that the transcripts were amplified when we used *B. sorghicola* genomic DNA but not when we used sorghum DNA (data not shown).

**Table 1:** Transcripts of *Bipolaris sorghicola* expressed during its growth in *Sorghum bicolor* plants.

Transcript	Length	Description	E-value in BLASTX search
Locus_4789_Transcript_7	2,225	Hex1 (Woronin body major protein)	2.00E-73
Locus_37669_Transcript_1	984	LysM domain-containing Intracellular hyphae protein	4.00E-15
Locus_38044_Transcript_1	694	Cross-pathway control protein 1	5.00E-24
Locus_36952_Transcript_1	1,012	Transcriptional activator hac1	3.00E-29
Locus_31150_Transcript_1	1,241	Endo-1,4-beta-xylanase 1	1.00E-53
Locus_38_Transcript_4	1,118	Acetylxylan esterase 2	7.00E-14
Locus_37767_Transcript_1	1,257	Sphingosine-1-phosphate lyase	1.00E-129
Locus_36040_Transcript_2	1,350	Cytochrome c peroxidase, mitochondrial	8.00E-159
Locus_37661_Transcript_1	494	Thioredoxin	1.00E-39
Locus_37730_Transcript_2	777	Mitochondrial peroxiredoxin PRX1	1.00E-58
Locus_37731_Transcript_5	2,561	Catalase-peroxidase	0
Locus_37936_Transcript_1	536	Superoxide dismutase [Cu-Zn]	5.00E-92
Locus_31331_Transcript_1	819	Putative sterigmatocystin biosynthesis protein stcT	6.00E-48
Locus_37968_Transcript_1	501	Uncharacterized protein C17G6.02c	6.00E-16
Locus_27055_Transcript_1	506	Translationally-controlled tumor protein homolog	2.00E-46
Locus_31947_Transcript_1	1,622	Elongation factor 1-alpha	0

### Identification of differentially Expressed Genes in Sorghum

Expression of sorghum transcripts was quantified by using reads aligned to unique locations on the reference genome. We adopted gene models annotated in Phytozome [18] and calculated RPKM values. To elucidate the transcripts that were unannotated in Phytozome, novel transcripts were identified by using the Cufflinks program [19] on the basis of the piling-up of aligned reads. We identified 9454 unannotated transcripts, with a maximum length of 27,400 bp, minimum length of 100 bp, average length of 1314 bp, and median length of 848 bp. Their RPKM values were calculated in the same way as for the annotated transcripts. Pathogen-specific regulation of gene expression was detected by using statistical testing (see Materials and Methods): 996 pathogen-induced and 163 pathogen-suppressed transcripts were observed. Here, we focus mainly on the pathogen-induced transcripts.

To obtain an overview of the transcriptome changes, we retrieved statistically over-represented Pfam families or domains in the pathogen-induced genes by using Fischer’s exact test with a false discovery rate (FDR) threshold of ≤0.1%. The domains of many defense-related proteins (Pkinase, WRKY, and Thaumatin) were over-represented among the pathogen-induced genes (Table S2).

### Plant Receptors

Because plant receptors possess diverse domain architecture, we grouped them on the basis of their Pfam domains and examined the numbers of pathogen-induced transcripts for each group (see Materials and Methods). We identified 933 sorghum receptors, including 443 receptor-like kinases (RLKs), 162 leucine-rich repeat (LRR) proteins, and 328 nucleotide-binding site (NBS)-LRR proteins. We further categorized the RLKs into subfamilies on the basis of their N-terminal domains: we found 222 LRR kinases, 50 lectin kinases, 66 self-incompatibility locus (S-locus) kinases, 3 lysine motif (LysM) kinases, 62 wall-associated kinases (WAKs), 2 PR5-like receptor kinases (PR5Ks), and 38 cysteine-rich RLKs (CRKs).

**Figure 2:**
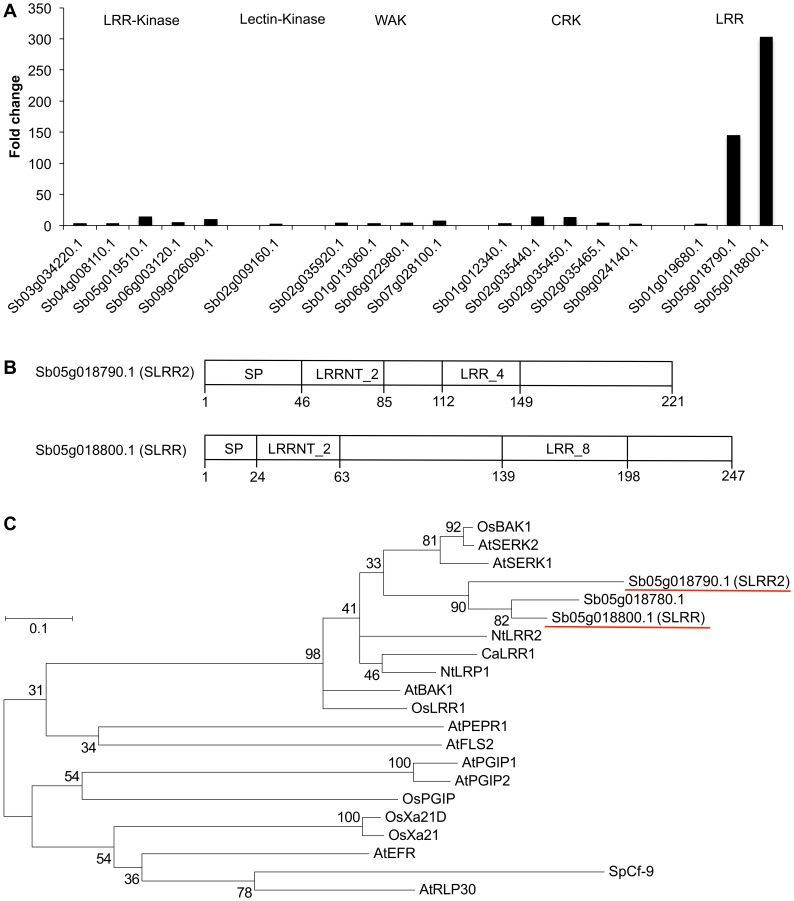
Pathogen (*Bipolaris sorghicola*)-induced genes encoding receptors in sorghum. (A) Fold changes of pathogen-induced transcripts for each family. RPKM fold changes at 24 h were calculated for infected samples compared with mock-infected samples. Genes with a fold change value of ≥3 were shown. To avoid division by 0, 1 was added to the RPKM values of mock-infected samples. Families of the receptors, shown on the top, were defined on the basis of the Pfam domain structures (See Materials and Methods). (B) Domain structures of the 2 receptors for which the genes were highly induced. Predicted structures included SP, signal peptide; LRRNT_2, Leucine rich repeat N-terminal domain (PF08263); LRR_4, Leucine Rich repeats (2 copies) (PF12799); and LRR_8, Leucine rich repeat (PF13855). (C) Phylogenetic tree of the 2 receptors for which the genes were particularly strongly induced, along with their homologous proteins. The amino acid sequences of LRRNT_2 domain (PF08263) of each protein were aligned by using ClustalW and the tree was created by using MEGA5. Red underlines show the 2 receptors for which the genes were particularly strongly induced. Abbreviations are as follows: Sb, *Sorghum bicolor*; Os, *Oryza sativa* (rice); At, *Arabidopsis thaliana*; Nt, *Nicotiana tabacum* (tobacco); Ca, *Capsicum annuum* (pepper); Sp, *Solanum pimpinellifolium* (currant tomato).

Among the 933 receptors, the genes encoding 73 receptors were pathogen induced (Figure 2A). Of these, the genes encoding 2 LRR proteins were induced with very high fold changes (>100) (Figure 2A). One of them, *Sb05g018800.1* (known as *SLRR*), is induced by another fungal pathogen [20]. The other one, *Sb05g018790.1*, is located adjacent to the *SLRR* gene in the genome; it shares significant sequence identity with SLRR (an E value of 1.4e-62 with 58.9% identity in the BLASTP search). On the basis of these characteristics, we called this second gene *SLRR2*. The structural details of SLRR and SLRR2 are shown in Figure 2B. Both proteins have LRR domains and signal peptides, but lack kinase and transmembrane domains (Figure 2B). These structural characteristics are similar to those of polygalacturonase-inhibiting proteins (PGIPs) and Xa21d, which are well-known LRR proteins involved in plant defense responses [21]. However, phylogenetic analysis showed that SLRR and SLRR2 were more closely related to the LRR domains of RLKs such as SERK1 and BAK1 (well-known RLKs involved in plant defense responses [21]) than to their structural analogs PGIP and Xa21d (Figure 2C).

### Genes for Signaling Cascades

The MAPK cascade and Ca^2+^ signaling act downstream of receptors [3]. The MAPK cascade consists of a MAPK kinase kinase (MAPKKK), MAPK kinase (MAPKK), and a MAPK. The Ca^2+^ signaling pathway consists of CDPKs (Ca^2+^-dependent PKs), CBL/CIPKs (calcineurin B-like/CBL-interacting PKs), and calmodulins (CaM). We identified 210 sorghum transcripts, consisting of 10 MAPKKKs, 9 MAPKKs, 19 MAPKs, 32 CDPKs, 14 CBLs, 35 CIPKs, and 91 CaMs (See Materials and Methods). The 210 transcripts corresponded to 191 genes.

Among the 191 genes, 13 were pathogen induced (Table 2). Four of these genes–2 for CDPK (*Sb06g026530* and *Sb04g002220*), one for a CIPK (*Sb02g042910*), and one for a CaM (*Sb02g024550*)–were also induced in our previous transcriptome study [13], in which gene expression at a relatively late stage of infection (7 days post-inoculation) was analyzed in the disease-susceptible cultivar BTx623 (Table 2). This induction in different cultivars at different infection stages supports the involvement of these 4 genes in the defense response. Phylogenetic tree analysis showed that Sb04g002220, Sb06g026530, and Sb02g042910 are putative orthologs of OsCPK4, OsCPK13, and OsCIPK29, respectively, in rice (Figure S1). No ortholog of CaM Sb02g024550 was detected by using this method (data not shown).

**Table 2:** Pathogen (*Bipolaris sorghicola*)-induced genes encoding proteins for signaling cascade in *Sorghum bicolor.*

Gene	Family	Fold change	
		SIL-05	BTx623^1^
Sb01g030680	MAPK	1.75	–
Sb04g031570	CDPK	1.22	–
Sb06g026530	CDPK	2.04	2.53
Sb04g002220	CDPK	2.33	1.63
Sb02g009790	CDPK	1.49	–
Sb02g042910	CIPK	1.51	9.67
Sb03g038380	CIPK	1.89	–
Sb07g003070	CaM	2.08	–
Sb02g024550	CaM	13.26	33.52
Sb05g020380	CaM	1.58	–
Sb1599s002010	CaM	1.16	–
Sb01g008460	CaM	1.28	–
Sb09g024040	CaM	1.08	–

1 data from our previous study [13].

### Transcriptional Factors

**Figure 3:**
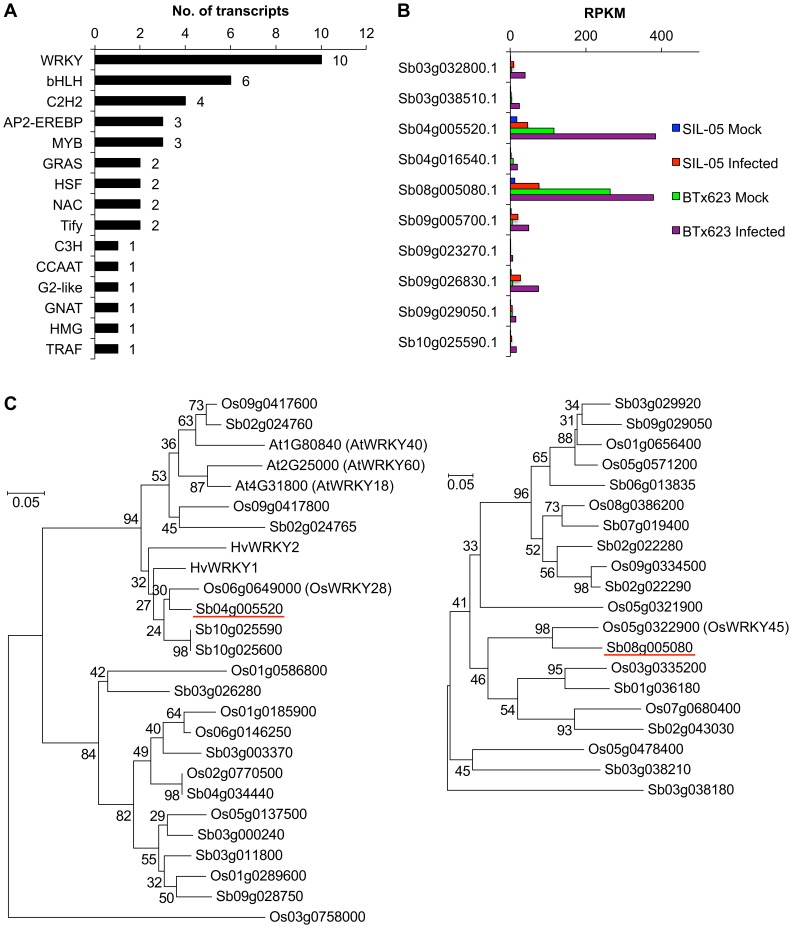
Pathogen (*Bipolaris sorghicola*)-induced genes encoding transcriptional factors in sorghum. (A) Number of pathogen-induced transcripts for each family. Families of the transcriptional factors (TFs), shown at left, were defined on the basis of a homology search in the Plant Transcription Factor Database [22]. (B) Expression of the 10 induced WRKY TFs. RPKMs of each transcript were compared in mock- and pathogen-infected leaves by using the disease-resistant cultivar SIL-05 in the early stages of infection (24 h) (this study) and a related cultivar, BTx623, at a relatively late infection stage (7 days) (our previous study [13]) (C) Phylogenetic tree of the 2 WRKY TFs for which the genes were expressed at relatively high levels. The amino acid sequences of WRKY domain (PF03106) of Sb08g005080 (red underline in right panel) and Sb04g005520 (red underline in left panel), and their best 10 BLAST hits in the Phytozome sorghum protein database [16] and the Rice Annotation Project (RAP) protein database (http://rapdb.dna.affrc.go.jp), were aligned by using ClustalW. Phylogenetic trees were constructed by using MEGA5. For Sb04g005520, HvWRKY1/2 and ATWRKY40/60 were also aligned. Abbreviations are as follows: Sb, *Sorghum bicolor*; Os, *Oryza sativa* (rice); Hv, *Hordeum vulgare*; At, *Arabidopsis thaliana*.

To identify pathogen-induced TFs, we searched the TFs in the sorghum genome by using a BLASTP search in PlnTFDB (the Plant Transcription Factor Database) [22] and examined the numbers of pathogen-induced ones. Among the 2568 TFs, 40 were pathogen-induced (Figure 3A).

WRKY is the TF family most commonly involved in transcriptional regulation associated with plant immune responses [23]. In fact, WRKY TFs were statistically over-represented among the pathogen-induced genes (Table S2) and constituted the largest number of all pathogen-induced TFs (Figure 3A). We also performed Fisher’s exact tests to find the TF binding sites (TFBSs) that were statistically over-represented among the pathogen-induced genes. WBOX-like TFBSs, which are the binding sites of WRKY family TFs, were over-represented (shown in bold type in Table S3).

In total, 10 WRKY TFs were pathogen-induced. These 10 WRKY TFs were also induced in our previous transcriptome study with BTx623 [13] (Figure 3B). Two WRKY TFs (Sb04g005520.1 and Sb08g005080.1) showed relatively high expression in both cultivars (Figure 3B). Phylogenetic tree analysis showed that Sb04g005520.1 and Sb08g005080.1 are putative orthologs of OsWRKY28 and OsWRKY45, respectively (Figure 3C). In addition, Sb04g005520.1 was highly related to the barley WRKY TFs HvWRKY1 and HvWRKY2 (Figure 3C).

### Downstream Responses

Activation of upstream signaling pathways leads to activation of defense mechanisms, including biosynthesis of phenylpropanoids (such as flavonoids) and lignin, generation of reactive oxygen species (ROS), and production of so-called pathogenesis-related (PR) proteins. Genes for flavonoid metabolism were pathogen-induced in *S. bicolor*; they included PAL (phenylalanine ammonia lyase), C4H (trans-cinnamate 4-monooxygenase), 4CL (4-coumarate:CoA ligase), CHS (chalcone synthase), and CHI (chalcone isomerase) (Table S3). Genes for proteins involved in the formation of lignin were also induced; they included CCR (cinnamoyl-CoA reductase), CAD (cinnamyl alcohol dehydrogenase), and dirigent protein (Table S4). In addition, several PR proteins were pathogen-induced and gave very high RPKM values (Table S5).

**Figure 4:**
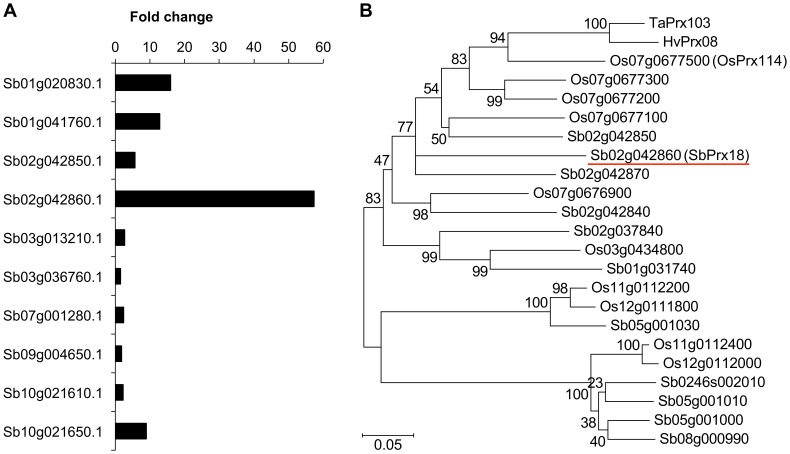
Pathogen (*Bipolaris sorghicola*)-induced genes encoding peroxidases in sorghum. (A) Pathogen-induced fold changes in expression. RPKM fold changes at 24 h were calculated for infected samples compared with those of mock-infected samples. To avoid division by 0, 1 was added to the RPKM values of mock-infected samples. (B) Phylogenetic tree of peroxidases for which the encoding genes were strongly induced. The amino acid sequences of peroxidase domain (PF00141) of Sb02g042860 (SbPrx18) (red underline) and its best 10 BLAST hits in the Phytozome sorghum protein database [16] and the Rice Annotation Project (http://rapdb.dna.affrc.go.jp) protein database, as well as the sequences of wheat TaPrx103 and barley HvPrx08, were aligned by using ClustalW. Phylogenetic tree was constructed by using MEGA5. Abbreviations are as follows: Sb, *Sorghum bicolor*; Os, *Oryza sativa* (rice); Hv, *Hordeum vulgare*; Ta, *Triticum aestivum*.

Peroxidases play important roles in defense. They are involved in the formation of lignin and both production and scavenging of ROS [24], [25]. Among the 153 sorghum genes encoding peroxidases that were identified on the basis of the Pfam domain, 10 genes were pathogen-induced; *Sb02g042860* was particularly highly induced (Figure 4A). Sb02g042860 is a class III peroxidase called SbPrx18 in PeroxiBase [26], and forms a peroxidase cluster with Sb02g042840, Sb02g042850, and Sb02g042870 on chromosome 2. Phylogenetic analysis showed that the cluster to which SbPrx18 belongs is closely related to a rice peroxidase cluster on chromosome 7 to which a well-characterized peroxidase, OsPrx114, belongs, suggesting that there is a close relationship between SbPrx18 and OsPrx114 (Figure 4B). In fact, SbPrx18 had the best BLAST hit to OsPrx114 in the Rice Annotation Project protein database (http://rapdb.dna.affrc.go.jp). In addition, SbPrx18 was closely related to wheat TaPrx103 and barley HvPrx08 (Figure 4B).

## Discussion

### Role of Genes of *B. sorghicola* Expressed during Infection of Sorghum

We identified 160 *B. sorghicola* transcripts that were expressed during growth of the fungus in sorghum. This is one of the few examples of successful transcriptome assembly in a pathogen during its growth in plants. We focused on 16 transcripts (Table 1) and confirmed by genomic PCR that they were of pathogen origin (data not shown). The 16 transcripts included genes potentially involved in growth of this fungus in sorghum, such as those encoding Hex1, LysM-domain-containing intracellular hyphae protein, 2 transcriptional factors, and plant-cell-wall-degrading enzymes (Table 1).

Hex1 is the major protein of the Woronin body, which is a septal-pore-associated organelle that is unique to filamentous ascomycetes. Chua et al. [27] reported the important cellular functions of Woronin bodies during the pathogenic phase of the rice blast fungus *Magnaporthe grisea.* They showed that mutation of the Hex1 gene in *M. grisea* leads to morphological and functional defects in the appressoria (infection structures) and delays host penetration; it subsequently disrupts invasive hyphal growth *in planta* and thus reduces pathogenicity. These data suggest that the Hex1 identified here is recruited by *B. sorghicola* to establish fungus penetration and subsequent growth in sorghum.

Production of LysM-domain-containing intracellular hyphae protein is induced in blast fungus during its growth in rice [8]. It has been proposed that fungal LysM-containing proteins suppress the host immunity triggered by chitin oligosaccharides [28]. Chitin oligosaccharides are breakdown products of fungal cell walls; they are released during invasion and act as triggers of host immunity. LysM-containing proteins suppress immunity through their involvement in the sequestration of chitin oligosaccharides. These findings suggest that the LysM-domain-containing intracellular hyphae protein identified here contributes to the suppression of host immunity.

The 2 identified TFs were cross-pathway control protein 1 (CpcA) and Hac1 (also called as HacA). CpcA is the central TF of the cross-pathway control system involved in the upregulation of genes for amino acid biosynthesis under conditions of amino acid limitation [29]. The importance of this gene has been reported in some pathogenic fungi. In *Aspergillus fumigatus*, mutation of the gene encoding CpcA leads to attenuation of virulence [30]. In *Leptosphaeria maculans*, CpcA regulates the production of sirodesmin PL, which is a secondary metabolite toxin [31]. HacA is the master transcriptional regulator of the unfolded protein response (UPR) [32]. The UPR balances the load of newly synthesized proteins with the folding capacity of the endoplasmic reticulum and allows cells to adapt to situations that disrupt this balance [33]. In the absence of HacA, *A. fumigatus* is unable to secrete high levels of proteins and has reduced virulence in mice [34]. These findings suggest that CpcA and HacA identified here are involved in the virulence of *B. sorghicola*.

The plant-cell-wall-degrading enzymes identified were endo-1,4-beta-xylanase and acetylxylan esterase. These enzymes are involved in the degradation of xylan, which is the major hemicellulose component of the cell wall in grasses [35], [36]. Many plant-pathogenic fungi produce a range of cell-wall-degrading enzymes that are probably involved in penetration and infection of host-plant tissues [37]. We therefore consider that expression of the endo-1,4-beta-xylanase and acetylxylan esterase identified here contributes to degradation of the sorghum cell wall and to the subsequent development of infection. In addition, endo-1,4-beta-xylanase is an effector protein that triggers defense response reactions in tobacco (*Nicotiana tabacum*) and tomato (*Solanum lycopersicum*). Recognition of the endo-1,4-beta-xylanase referred to as ethylene-inducing xylanase (EIX) in tomato requires the receptor-like proteins LeEIX1 and LeEIX2. Although genes homologous to those encoding LeEIX1 and LeEIX2 in sorghum (Top 5 BLAST hits) were expressed at very low levels (RPKM <1) (data not shown), it is possible that the endo-1,4-beta-xylanase identified here acts as an effector protein in sorghum infected with *B. sorghicola*.

We were thus able to identify potential genes for infection despite the unavailability of the *B. sorghicola* genome sequence. Further characterization of these genes will help to elucidate the mechanism of infection with *B. sorghicola* in target leaf spot disease.

### Defense-oriented Reprogramming of the Sorghum Transcriptome in the Early Stages of Infection

There are many reports of changes in the transcriptomes of host plants as a result of infection with pathogens [4], [38], [39]. In the interaction of sorghum and *B. sorghicola*, pathogen infection dramatically changes the expression of genes that encode enzymes involved in the sorghum-specific secondary metabolic network, suggesting that sorghum-specific phytochemicals are synthesized in the late stages of infection [13]. Here, we focused on transcriptome changes in the disease-resistant cultivar SIL-05 in the early stages of infection with *B. sorghicola*. Global transcriptome analysis revealed distinct expression among duplicated genes (Figures 2 to 4). Below, among homologs annotated in the sorghum genome, we discuss the characteristics of the particular genes that showed induction of expression upon infection with *B. sorghicola*. We focus especially on LRR family receptors, WRKY transcription factors, peroxidases, and Ca^2+^ signaling proteins.

### (i) SLRR2, a New Member of the Simple eLRR Domain Protein Family, Induced in the Defense Response

Among the genes for 933 receptor candidates in the sorghum genome, those encoding 2 LRR proteins were particularly strongly induced (Figure 2A). One was SLRR. The gene encoding this protein has been isolated as being induced after inoculation of sorghum with the fungal pathogen *Colletotricum graminicola* [20]. The other was SLRR2, which is a homolog of SLRR.

Recently, some groups have proposed that SLRR belongs to an LRR subfamily called the simple eLRR domain protein family [40]–[43]. Although members of the family are structurally similar to the LRR-only receptor PGIP, they show greater sequence identity to the LRR domains of receptor-like kinases such as SERK1, SERK2, and BAK1. Our results supported these findings (Figure 2B and C). We found the same characteristics in SLRR2 (Figure 2B and C). Two groups have reported that OsLRR1–a member of the family in rice–enhances disease resistance when ectopically expressed in Arabidopsis and Chinese cabbage [40], [43]. We therefore conclude that SLRR2 is a new member of the simple eLRR domain protein family and that SLRR and SLRR2 are involved in the defense response.

Moreover, we predicted that both SLRR and SLRR2 would have a signal peptide. This suggests their entrance to the protein translocation pathway. In fact, some members of the family have been found in various cellular locations in experiments using proteins of the simple eLRR domain protein family fused with green fluorescent protein [40], [42], [44]. Further investigations will reveal the cellular locations of SLRR and SLRR2.

The molecular mechanisms of SLRR and SLRR2 involvement in the plant defense response are unclear. Some members of the family interact with HIR1, which plays an important role in the hypersensitivity response [40], [43], [45], [46]. For example, in rice, OsHIR1 interacts with OsLRR1, which is a member of the simple eLRR domain protein family. We identified Sb07g019760 in sorghum as a putative ortholog of OsHIR1, but its expression did not significantly change for at least 24 h after infection with *B. sorghicola* (data not shown). As OsHIR1 accumulates gradually for 6 days after inoculation with *Xanthomonas oryzae* [45], Sb07g019760 might be expressed at a relatively late stage after infection and might thus play a role in the hypersensitive response. Further investigation is also required to elucidate whether SLRR and SLRR2 interact with HIR1.

### (ii) WRKY TFs, Including OsWRKY28 and OsWRKY45 Orthologs, are Induced in the Defense Response

We found several pieces of evidence that WRKY TFs were involved in the defense response (Figure 3A, and Table S2 and S3). From these results, we consider that WRKY TFs are the main TFs in the *B. sorghicola*-triggered defense response. We found that 10 WRKY genes were induced in *S. bicolor*; these 10 genes were also induced in our previous study with BTx623 [13]. The induction of the 10 genes in different cultivars at different infection stages supports their involvement in the defense response. Among them, *Sb04g005520* and *Sb08g005080* showed relatively high expression in both SIL-05 and BTx623 (Figure 3B).

Sb04g005520 is a putative ortholog of rice OsWRKY28. OsWRKY28 and its putative orthologs HvWRKY1 and HvWRKY2 regulate basal defense in rice and barley, respectively [47], [48]. These findings support the importance of Sb04g005520 in the defense response in sorghum. It has been proposed that OsWRKY28, and HvWRKY1 and -2 are negative regulators. Delteil et al. [47] hypothesized that *Magnaporthe oryzae* blocks host defenses via the control of OsWRKY28. This hypothesis is based on the fact that *M. oryzae* infection induces OsWRKY28 expression, thus suppressing PR gene expression. In fact, it has been proposed that pathogens possess mechanisms for suppressing plant immunity [49]. Although experimental evidence is needed to determine whether Sb04g005520 also acts as a negative regulator, induction of the genes encoding WRKY TFs of the OsWRKY28/Sb04g005520 family may be a major mechanism used by pathogens to suppress plant immunity.

Sb08g005080 is an ortholog of rice OsWRKY45. Shimono et al. [50], [51] showed that overproduction of OsWRKY45 induces extremely strong resistance to the fungal pathogen *M. grisea* and the bacterial pathogen *X. oryzae*, with minor growth retardation. Because of these agronomically important traits, OsWRKY45 is a promising candidate for use in the development of multidisease-resistant crops. These ortholog data suggest that Sb08g005080 is important for resistance to *B. sorghicola* in sorghum and might have agricultural applications. Collectively, the 10 WRKY TFs–especially Sb04g005520 and Sb08g005080–are important for the defense response in sorghum.

*Sb04g005520* and *Sb08g005080* were highly expressed in both the resistant cultivar SIL-05 and the susceptible cultivar BTx623 (Figure 3B). However, their respective orthologous proteins (OsWRKY28 and OsWRKY45) have opposite functions, namely suppressing plant immunity or promoting plant defense response. This functional difference might be related to differences in the degrees of resistance and susceptibility in the sorghum plant.

### (iii) SbPrx18 Peroxidase is Potentially a Key Player in the Defense Response

We found that typical downstream response genes in sorghum were pathogen-induced (Tables S4 and S5), suggesting that downstream responses had already occurred in the early stage of infection. We focused on the strong induction of the class III peroxidase Sb02g042860 (SbPrx18) by the pathogen (Figure 4A). Plant class III peroxidases are involved in numerous responses related to pathogen resistance, including the control of H_2_O_2_ levels and lignin formation [24], [52]. The diversity of the processes catalyzed by peroxidases, as well as the large number of their genes, suggests the possibility of functional specialization of each isoform [24]. Specific peroxidase isoforms are induced during particular processes or in particular locations within plants [53], [54]. Notably, among the 153 homologous genes (including those encoding class I and II peroxidases) in the sorghum genome, the expression of that encoding SbPrx18 was particularly strongly induced by *B. sorghicola*. This result suggests that SbPrx18 is the main peroxidase for the defense response in sorghum.

Moreover, we showed that SbPrx18 is highly related to the rice peroxidase OsPrx114 (Figure 4B). OsPrx114 (previously referred to as PO-C1) is a putative lignin-forming peroxidase, and its production is strongly induced after inoculation with rice bacterial blight pathogen [55]–[57]. Recently, Wally and Punja [58] reported that overproduction of OsPrx114 increases resistance to necrotrophic pathogens in carrot through increased PR transcript accumulation, rapid removal of H_2_O_2_ during oxidative burst response, and enhanced lignin formation. These ortholog data confirm the importance of SbPrx18 in the defense response in sorghum. In addition, Wally and Punja mentioned that the highest degree of amino acid identity to OsPRx114 was found in wheat peroxidase TaPrx103 and barley HvPrx08. In fact, SbPrx18 was also highly related to these peroxidases (Figure 4B). TaPrx103 and HvPrx08 are involved in disease resistance [59]–[61]. These findings suggest that recruitment of peroxidases from the family to which SbPrx18 belongs is a common disease resistance mechanism in poaceous plants.

### (iv) Ca^2+^ Signaling Proteins are Potentially Involved in the Defense Response

We demonstrated the induction of Ca^2+^ signaling genes in the defense response. The genes were those encoding 2 CDPKs (Sb04g002220 and Sb06g026530), 1 CIPK (Sb02g042910), and 1 CaM (Sb02g024550). One of the CDPKs, Sb04g002220, is the putative ortholog of OsCPK4 in rice (Figure S1). OsCPK4 is transcriptionally activated in response to inoculation with the arbuscular mycorrhizal fungus *Glomus intraradices* [62]. These ortholog data suggest that Sb04g002220 is involved in responses to both pathogenic and symbiotic fungi. The other CDPK, Sb06g026530, is the putative ortholog of rice OsCPK13 (previously called CDPK-7) (Figure S1). When ectopically produced in sorghum, OsCPK13 induces cell death, accumulation of PR proteins, and upregulation of some defense genes [63], [64]. Our findings confirm the importance of Sb06g026530 in the defense response. The CIPK Sb02g042910 is the putative ortholog of OsCIPK29 (Figure S1). The report that OsCIPK29 is induced by stresses such as drought, salinity stress, polyethylene glycol (PEG), and abscisic acid (ABA) [65] suggests that Sb02g042910 could be involved in multiple stress responses. CaM Sb02g024550 was induced at relatively high levels (Table 2). Although we failed to assign an ortholog to this gene, its relatively strong induction suggests its involvement in the defense response. Further characterization of these genes should help to elucidate downstream signaling of receptors in sorghum innate immunity.

We focused here on genes derived from sorghum and fungus in association with the roles of known homologs and cascades in the defense mechanism. However, the roles that most of the genes identified play in plant–pathogen interactions are still unknown. Integration of the genome, transcriptome, proteome, and metabolome will generate many new leads and hypotheses in regard to comprehensive understanding of plant–pathogen interactions in the future.

### Conclusions

We simultaneously analyzed the transcriptomes of sorghum and *B. sorghicola* by using RNA-seq in combination with *de novo* transcriptome assembly. Genes in *B. sorghicola* that were involved in growth of the fungus on sorghum, and genes in sorghum that were involved in the defense response, were identified. Expression of these defense-related genes was particularly strongly induced among paralogs annotated in the sorghum genome. Further characterization of these genes will help to elucidate the mechanism of sorghum – *B. sorghicola* interaction. Our strategy is useful for examining plant–microorganisms interactions in the absence of genomic sequence information.

## Materials and Methods

### Plant and Fungal Material Preparation, and mRNA Sequencing

Sorghum (*Sorghum bicolor* L. Moench) SIL-05, which is resistant to target leaf spot, was used for mRNA seq. Four seeds were sterilized, placed onto Kumiai-Ryujyou-Baido soil (Kureha Chemical, Tokyo, Japan) in each plant box (5×5×15 cm high) and incubated in a chamber for 7 days under 16 h light and 8 h dark at 28°C. *Bipolaris sorghicola* isolate BC-24 (MAFF number 511379) was used as the inoculum. The BC-24 strain was grown on vegetable juice (Campbell V8) agar for 10 days in the dark at 25°C and then placed under UV light, where it was kept for 10 days to induce conidia formation. Conidia were harvested in 0.01% Tween-20, and the concentration of suspensions was adjusted to 4×10^5^ conidia/ml. On day 7 (about the 2- or 3-leaf stage) the sorghum plants were sprayed with 1 ml of this suspension per plant box. For the negative control, 0.01% Tween-20 solution was used. The plants were sampled at 0, 12, and 24 h after inoculation. Four plants per point were collected, immediately frozen in liquid nitrogen, and mixed to minimize the effect of transcriptome variability among individual plants. Total RNA was extracted with RNeasy (Qiagen, Hilden, Germany). The total RNA was subjected to further analysis. mRNA-seq library construction and sequencing were performed at Hokkaido System Science Co., Ltd. (Sapporo, Japan) in accordance with the manufacturer’s instructions by using an Illumina (San Diego, CA) TruSeq RNA Sample Prep Kit and 100-bp single-end protocol on a HiSeq2000 system (Illumina). All primary mRNA sequence read data were submitted to the DNA Data Bank of Japan (DDBJ: DRA000948).

### Preprocessing and Mapping of Illumina Reads

In total, we obtained 39,052,083 single-end reads 100 bp long from leaves before infection (0h), 30,689,318 from mock-infected leaves at 12 h, 36,462,229 from mock-infected leaves at 24 h, 32,911,638 from pathogen-infected leaves at 12 h, and 33,373,183 from pathogen-infected leaves at 24 h. We used an in-house program to trim out low-quality bases (<Q15) from both the 5′- and 3′- ends of the reads until a stretch of 3 bp or more of high-quality (≥Q15) bases appeared. Contaminating Illumina adapter sequences were also trimmed out by using Cutadapt version 1.0 [66]. The reads were aligned to the sorghum reference genome of the BTx623 cultivar [11] by using Bowtie 2 version 2.0.0 beta7 [67], SAMtools version 0.1.18 [68], and TopHat version 2.0.4 [69]. A gene transfer format (GTF) file provided by Phytozome [18] was supplied using the -G option; default values were used for other parameters.

### Retrieving Pathogen Transcripts

Reads that were unaligned to the reference genome were assembled by using Velvet version 1.2.03 [70] and Oases version 0.2.05 [14] with the parameters recommended in the “For impatient people” section of the Oases Manual version 0.2 (http://www.ebi.ac.uk/~zerbino/oases/OasesManual.pdf). Oases is designed to heuristically assemble RNA-seq reads in the absence of a reference genome [14]. Oases implements the multiple k-mer method, in which multiple individual k-mer assemblies are merged into a final assembly. This method yields an optimal overall assembly covering wide transcript expression. For individual k-mer assemblies, we used k-mer lengths from every odd number, starting from 21 and ending at 51.

To remove redundant transcripts, the assembled contigs were processed by using CD-HIT-EST version 4.5.4 [71] with default parameters. Redundant contigs that were entirely aligned to longer sequences with an identity threshold of 90% were removed. The contigs were aligned again to the sorghum reference genome by using Blat version 34 [72] with the default parameters; contigs that were aligned with a coverage threshold of ≥50% were removed. To quantify the expression of each contig, the reads used for the assembly were aligned back to the contigs by using Bowtie version 0.12.7. By using an in-house program, RPKM values were calculated with reads that were aligned to unique loci (predicted by Oases). To remove contaminating transcripts from non-*B. sorghicola* organisms, we first extracted the transcripts that were expressed (RPKM >0) in *B. sorghicola*-infected leaves but not expressed (RPKM = 0) in the leaves before infection. Second, we performed a BLASTX search of the NCBI non-redundant database with an E-value threshold of 1E-03 and a subject-coverage threshold of ≥50% using NCBI BLAST+ version 2.2.26 [73]; we extracted only those transcripts with top hits to proteins from Pleosporales fungi (NCBI Taxonomy ID: 92860). For functional annotation of each contig, we performed a BLASTX search against RefSeq [16] or the Swiss-Prot database [17] with an E-value threshold of 1E-03 by using BLAST+; we retrieved 160 transcripts with hits. For the RefSeq database, we used only data with the status code REVIEWED or VALIDATED. Details of assembled transcripts of *B. sorghicola* are listed (Table S6).

### Expression Analysis of Sorghum Genes

We used an in-house program to calculate RPKM values for each transcript annotated by Phytozome [18], using only those reads that were aligned to unique locations in the reference genome. Cufflinks version 2.0.2 was used to predict transcripts by the piling-up of aligned reads. Unannotated transcripts, which did not overlap with the annotated transcripts, were screened by comparison with the Phytozome annotation. RPKM values of the unannotated transcripts were calculated as for the annotated transcripts. For functional annotation of the unannotated transcripts, BLASTX searches against RefSeq [16] or the Swiss-Prot database [17] were performed with an E-value threshold of 1E-03 by using BLAST+, and the description of the top hit was assigned as a function. Annotated and unannotated transcripts differentially expressed between mock-infected and infected leaves, and between before and after infection, were detected by using a G-test with a 0.1% FDR, as previously described [13]. We considered transcripts that met the following criteria to be pathogen-induced or suppressed: (i) differential expression between mock-infected and infected leaves at both 12 and 24 h; and (ii) differential expression at both 12 and 24 h when compared with before infection.

### Retrieving Statistically Over-represented Pfam Domains and TFBSs

We performed Fischer’s exact test with an FDR threshold of ≤0.1% using R version 2.13.0 (http://www.R-project.org). We detected over-represented Pfam domains [74] or TFBSs in the PLACE database [75] in the pathogen-induced genes when compared with all other differentially expressed genes that did not meet the criteria described above.

### Categorization of Sorghum Genes

We categorized sorghum genes mainly on the basis of Pfam domains [74], NCBI Conserved Domains [76], or PANTHER families [77] of proteins encoded by them. The amino acid sequences of annotated transcripts were downloaded from Phytozome, and those of the unannotated transcripts were predicted by using the perl script “transcripts_to_best_scoring_ORFs.pl,” which is utility software of Trinity [9] version r2012–04–27. Pfam domains were predicted from the amino acid sequences by using PfamScan (ftp://ftp.sanger.ac.uk/pub/databases/Pfam/Tools) with an E-value threshold of 1E-03. NCBI Conserved Domains were predicted by using the NCBI Web CD-Search Tool (http://www.ncbi.nlm.nih.gov/Structure/bwrpsb/bwrpsb.cgi) with default parameters. PANTHER families were assigned by using InterProScan 5 Release Candidate 2 [78] with the parameters “-f tsv -t p -dp –goterms.”

Receptors were categorized on the basis of their Pfam domains. We categorized them into RLKs, LRR proteins, and NBS-LRR proteins. We identified 433 RLKs as proteins that had Pkinase (PF00069) or Pkinase_Tyr (PF07714) domains at their C-termini and receptor domains such as LRR at their N-termini. We further categorized the RLKs into subfamilies on the basis of their N-terminal domains. We identified 222 LRR kinases with LRR domains (PF08263, PF00560, PF12799, PF13516, PF13504, or PF13855), 50 lectin kinases with Lectin_legB (PF00139), 66 S-locus kinases with S_locus_glycop (PF00954), 3 LysM kinases with LysM (PF01476), 62 WAKs with WAK-related domains (PF13947 or PF14380), 2 PR5K with Thaumatin (PF00314), and 38 CRKs with Stress-antifung (PF01657). We identified 162 LRR proteins as having only LRR domains (PF08263, PF00560, PF12799, PF13516, PF13504, or PF13855), and we identified 328 NBS-LRR proteins as those with NB-ARC (PF00931).

Downstream signaling genes were categorized on the basis of their Pfam domains, NCBI Conserved Domains, and PANTHER families. MAPKs were identified as proteins with STKc_TEY_MAPK_plant (cd07858) or STKc_TDY_MAPK_plant (cd07859), MAPKKs as those with PKc_MAPKK_plant_like (cd06623), and MAPKKKs as those with STKc_MAPKKK (cd06606) or STKc_MEKK1_plant (cd06632). We categorized Ca^2+^ signaling proteins into calcineurin, CDPK, CIPK, and CaM. Calcineurins, CDPKs, and CIPKs were identified as proteins with CALCINEURIN B (PTHR23056), EFh (cd00051) in addition to Pkinase (PF00069) or Pkinase_Tyr (PF07714), and CIPK_C (cd12195), respectively. For identification of CaMs, we first screened all proteins with EFh (cd00051) apart from calcineurins, CDPKs, and CIPKs. We then manually extracted CaMs from them on the basis of their functional annotations.

Peroxidases were identified as proteins with a Pfam domain peroxidase (PF00141). Families of TFs were assigned by a BLASTP search against PlnTFDB using BLAST+ with an E-value threshold of 1E-03 and a subject-coverage threshold of 80%.

### Schematic Representation of Domain Structures

Pfam domains and transmembrane helices were predicted by using Pfam (http://pfam.sanger.ac.uk) [74] and TMHMM Server v. 2.0 (http://www.cbs.dtu.dk/services/TMHMM) [79], respectively, with default parameters. Signal peptides were predicted by using Signal-3L (http://www.csbio.sjtu.edu.cn/bioinf/Signal-3L) [80] and PrediSi (http://www.predisi.de.) [81] with default parameters.

### Phylogenetic Analysis

Amino acid sequences were aligned by using CLUSTALW [82]. Phylogenetic trees were constructed by the neighbor joining method implemented in MEGA5 [83] with the options “Poisson model” for “Model/Method,” “Uniform rates” for “Rates among Sites,” and “Complete deletion” for “Gaps/Missing Data Treatment.” The confidence for each branching node was assessed by bootstrap analysis with 1000 replica [84]. The amino acid sequences of sorghum, rice, and Arabidopsis were downloaded from the Phytozome sorghum protein database [18], the Rice Annotation Project (RAP) protein database (http://rapdb.dna.affrc.go.jp), and The Arabidopsis Information Resource (TAIR) database [85] respectively.

## Supporting Information

Figure S1 **Phylogenetic trees of genes for downstream signaling.** Three genes (red underlines) were analyzed. The amino acid sequences of Pkinase domain (PF00069) of Sb04g002220 (A), Sb06g026530 (B), and Sb02g042910 (C), with their best 10 BLAST hits in the Phytozome sorghum protein database [16] and the Rice Annotation Project protein database (http://rapdb.dna.affrc.go.jp), were aligned by using ClustalW. Phylogenetic trees were constructed by using MEGA5. Abbreviations are as follows: Sb, *Sorghum bicolor*; Os, *Oryza sativa* (rice).
(PDF)

Table S1 **Alignment of sequenced reads.**
(PDF)

Table S2 **Statistically over-represented Pfam domains in pathogen (** ***Bipolaris sorghicola*** **)-induced genes.**
(PDF)

Table S3 **Statistically over-represented transcription factor binding sites (TFBSs) in pathogen (** ***Bipolaris sorghicola*** **)-induced genes.**
(PDF)

Table S4 **Pathogen (** ***Bipolaris sorghicola*** **)-induced genes in** ***Sorghum bicolor*** **encoding enzymes for secondary metabolites.**
(PDF)

Table S5 **Pathogen (** ***Bipolaris sorghicola*** **)-induced genes in** ***Sorghum bicolor*** **encoding pathogenesis-related (PR) proteins.**
(PDF)

Table S6 **Assembled transcripts of** ***Bipolaris sorghicola.***
(XLS)
