# Supplementary figures and images for: Simultaneous Transcriptome Analysis of Sorghum and Bipolaris sorghicola by Using RNA-seq in Combination with De Novo Transcriptome Assembly
Source: PLoS One. 2013 Apr 30;8(4):e62460. doi: 10.1371/journal.pone.0062460 (PMC3640049; doi:10.1371/journal.pone.0062460)

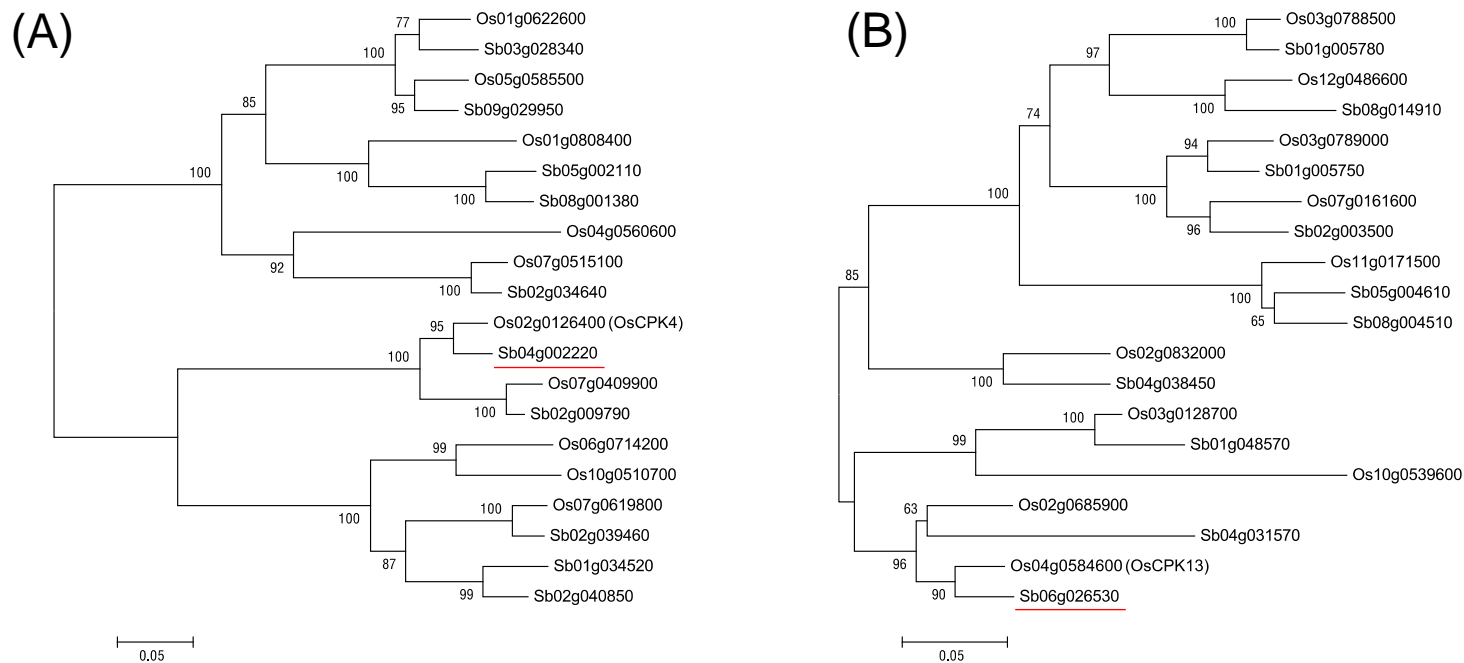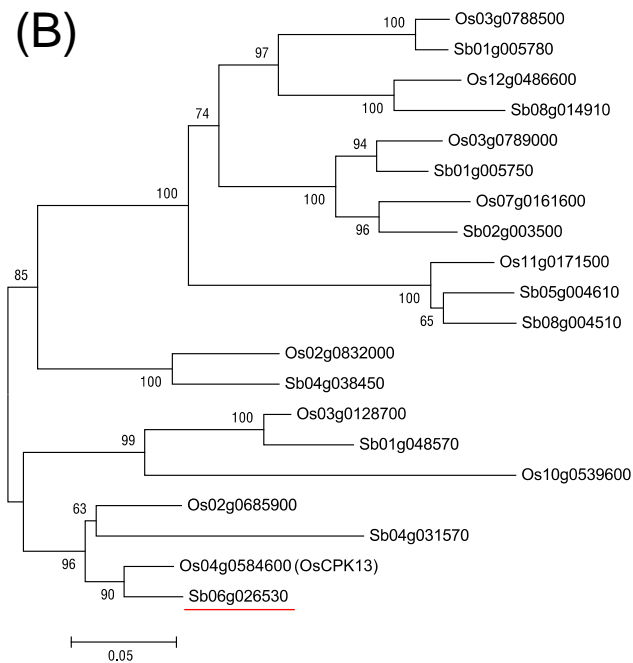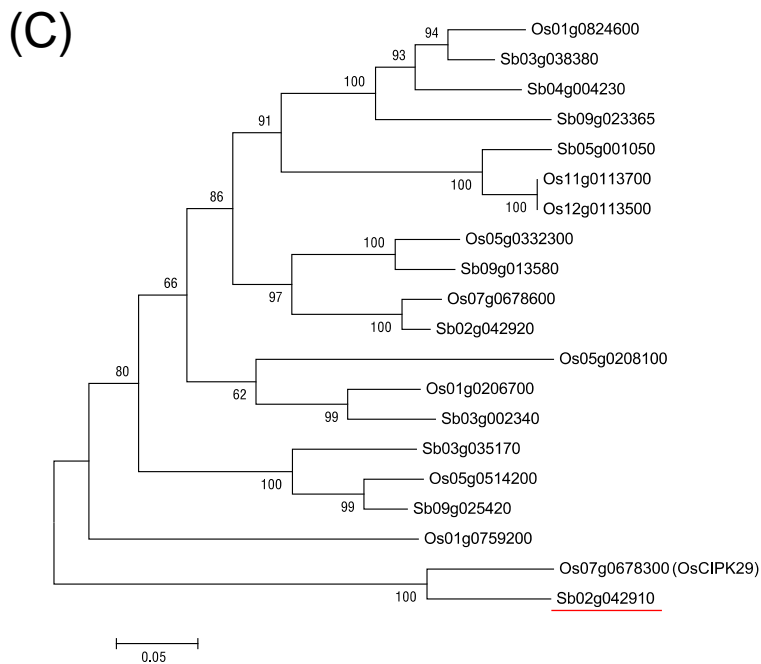

Figure S1

Supplement: Figure S1 — Phylogenetic trees of genes for downstream signaling. Three genes (red underlines) were analyzed. The amino acid sequences of Pkinase domain (PF00069) of Sb04g002220 (A), Sb06g026530 (B), and Sb02g042910 (C), with their best 10 BLAST hits in the Phytozome sorghum protein database [16] and the Rice Annotation Project protein database (http://rapdb.dna.affrc.go.jp), were aligned by using ClustalW. Phylogenetic trees were constructed by using MEGA5. Abbreviations are as follows: Sb, Sorghum bicolor; Os, Oryza sativa (rice). (PDF) [file pone.0062460.s001.pdf]
