# Supplementary material for: Simultaneous Transcriptome Analysis of Sorghum and Bipolaris sorghicola by Using RNA-seq in Combination with De Novo Transcriptome Assembly
Source: PLoS One. 2013 Apr 30;8(4):e62460. doi: 10.1371/journal.pone.0062460 (PMC3640049; doi:10.1371/journal.pone.0062460)
Supplement: Table S1 — Alignment of sequenced reads. (PDF) [file pone.0062460.s002.pdf]

Table S1 Alignment of sequenced reads

Sample	Time	No. of reads	Unique non-junction hit	%	Unique junction hit	%	Multi hit	%	Unaligned	%
control	0h	38,945,070	24,812,328	63.7	11,107,457	28.5	1,162,174	3.0	1,863,111	4.8
mock	12h	30,520,120	19,350,912	63.4	8,610,767	28.2	1,029,141	3.4	1,529,300	5.0
mock	24h	36,380,811	23,269,440	64.0	10,487,302	28.8	937,600	2.6	1,686,469	4.6
infected	12h	32,786,461	20,924,199	63.8	9,275,130	28.3	1,028,299	3.1	1,558,833	4.8
infected	24h	33,277,817	20,948,479	63.0	9,792,515	29.4	924,214	2.8	1,612,609	4.8
