# Supplementary material for: Simultaneous Transcriptome Analysis of Sorghum and Bipolaris sorghicola by Using RNA-seq in Combination with De Novo Transcriptome Assembly
Source: PLoS One. 2013 Apr 30;8(4):e62460. doi: 10.1371/journal.pone.0062460 (PMC3640049; doi:10.1371/journal.pone.0062460)
Supplement: Table S2 — Statistically over-represented Pfam domains in pathogen ( Bipolaris sorghicola )-induced genes. (PDF) [file pone.0062460.s003.pdf]

Table S2

Statistically over-represented Pfam domains in pathogen (*Bipolaris sorghicola*)-induced genes

| Pfam domain | Function | Number of genes |
|---|---|---|
| Pkinase | Protein kinase domain | 65 |
| p450 | Cytochrome P450 | 29 |
| ABC_tran | ATP-binding domain of ABC transporters | 15 |
| B_lectin | D-mannose binding lectin | 14 |
| GST_N_3 | Glutathione S-transferase, N-terminal domain | 12 |
| GST_C | Glutathione S-transferase, C-terminal domain | 11 |
| Thaumatin | Thaumatin | 11 |
| S_locus_glycop | S-locus glycoprotein family | 10 |
| WRKY | WRKY DNA -binding domain | 10 |
| Stress-antifung | Domain of unknown function | 9 |
| Lectin_legB | Leguminous lectin family | 9 |
| Glyco_hydro_18 | Glycoside hydrolase family 18 | 7 |
| Glyco_hydro_19 | Glycoside hydrolase family 19 | 7 |
| Chal_sti_synt_C | Chalcone and stilbene synthases, C-terminal domain | 7 |
| Chal_sti_synt_N | Chalcone and stilbene synthases, N-terminal domain | 7 |
| DUF4009 | Domain of unknown function | 7 |
| PDR_assoc | Plant PDR ABC transporter associated | 6 |
| Bet_v_1 | Bet v I allergen | 6 |
| Chitin_bind_1 | Carbohydrate-binding module | 6 |
| Methyltransf_2 | O-methyltransferase | 6 |
