# Supplementary material for: Simultaneous Transcriptome Analysis of Sorghum and Bipolaris sorghicola by Using RNA-seq in Combination with De Novo Transcriptome Assembly
Source: PLoS One. 2013 Apr 30;8(4):e62460. doi: 10.1371/journal.pone.0062460 (PMC3640049; doi:10.1371/journal.pone.0062460)
Supplement: Table S3 — Statistically over-represented transcription factor binding sites (TFBSs) in pathogen ( Bipolaris sorghicola )-induced genes. (PDF) [file pone.0062460.s004.pdf]

Table S3 Statistically over-represented TFBSs in pathogen (*Bipolaris sorghicola*)-induced genes

TFBS in PLACE database	Number	%
-300ELEMENT	314	31.5
ABRELATERD1	478	48
ABRERATCAL	412	41.4
ACGTATERD1	650	65.3
ACGTCBOX	91	9.1
ACGTOSGLUB1	72	7.2
ARR1AT	884	88.8
ASF1MOTIFCAMV	539	54.1
BIHD1OS	591	59.3
BOXLCOREDCPAL	238	23.9
CAATBOX1	880	88.4
CACTFTPPCA1	922	92.6
CGACGOSAMY3	493	49.5
CURECORECR	638	64.1
DOFCOREZM	918	92.2
DPBFCOREDCDC3	538	54
EBOXBNNAPA	810	81.3
EECCRCAH1	510	51.2
ELRECOREPCRP1	274	27.5
GATABOX	818	82.1
GT1CONSENSUS	844	84.7
GTGANTG10	890	89.4
HEXMOTIFTAH3H4	168	16.9
MYB2CONSENSUSAT	461	46.3
MYBCORE	656	65.9
MYCCONSENSUSAT	810	81.3
P1BS	108	10.8
POLASIG3	400	40.2
POLLEN1LELAT52	777	78
QELEMENTZMZM13	175	17.6
RAV1AAT	587	58.9
RHERPATEXPA7	452	45.4
ROOTMOTIFTAPOX1	665	66.8
RYREPEATBNNAPA	311	31.2
RYREPEATGMGY2	190	19.1
RYREPEATLEGUMINBOX	247	24.8
SEF1MOTIF	211	21.2
SEF4MOTIFGM7S	496	49.8
SORLIP1AT	605	60.7
SORLREP3AT	48	4.8
T/GBOXATPIN2	152	15.3
TATABOX2	256	25.7
TATABOX3	114	11.4
TATABOX4	246	24.7
TATABOX5	517	51.9
TATABOXOSPAL	244	24.5
TATAPVTRNALEU	123	12.3
TGACGTVMAMY	168	16.9
WBBOXPCWRKY1	213	21.4
WBOXATNPR1	655	65.8
WBOXHVISO1	558	56
WBOXNTCHN48	358	35.9
WBOXNTERF3	764	76.7
WRKY71OS	903	90.7
