# Supplementary material for: Simultaneous Transcriptome Analysis of Sorghum and Bipolaris sorghicola by Using RNA-seq in Combination with De Novo Transcriptome Assembly
Source: PLoS One. 2013 Apr 30;8(4):e62460. doi: 10.1371/journal.pone.0062460 (PMC3640049; doi:10.1371/journal.pone.0062460)
Supplement: Table S4 — Pathogen ( Bipolaris sorghicola )-induced genes in Sorghum bicolor encoding enzymes for secondary metabolites. (PDF) [file pone.0062460.s005.pdf]

Table S4

Pathogen (*Bipolaris sorghicola*)-induced genes in *Sorghum bicolor* encoding enzymes for secondary metabolites

		RPKM			
Family	Transcript	Conrtol	Mock	Infected	Annotation
PAL	Sb06g022750.1	1.33	2.04	4.36	Phenylalanine ammonia-lyase OS=Triticum aestivum GN=PAL PE=3 SV=1
PAL	Sb04g026560.1	1.55	4.70	6.08	Phenylalanine ammonia-lyase OS=Oryza sativa subsp. indica GN=ZB8 PE=3 SV=1
C4H	Sb02g010910.1	13.10	34.15	68.03	Trans-cinnamate 4-monooxygenase OS=Glycyrrhiza echinata GN=CYP73A14 PE=2 SV=1
C4H	Sb03g038160.1	0.29	1.91	3.30	Trans-cinnamate 4-monooxygenase OS=Catharanthus roseus GN=CYP73A4 PE=2 SV=1
C4H	Sb04g017460.1	0.02	0.00	0.34	Trans-cinnamate 4-monooxygenase OS=Glycyrrhiza echinata GN=CYP73A14 PE=2 SV=1
4CL	Sb04g005210.1	14.67	21.35	38.39	Probable 4-coumarate--CoA ligase 3 OS=Oryza sativa subsp. japonica GN=4CL3 PE=2 SV=1
4CL	Sb01g048200.1	4.41	5.89	14.43	4-coumarate--CoA ligase-like 4 OS=Oryza sativa subsp. japonica GN=4CLL4 PE=2 SV=1
4CL	Sb07g007810.1	3.27	6.25	19.57	Probable 4-coumarate--CoA ligase 1 OS=Oryza sativa subsp. japonica GN=4CL1 PE=2 SV=2
CHS	Sb05g020230.1	0.05	0.37	46.81	Chalcone synthase 6 OS=Sorghum bicolor GN=CHS6 PE=3 SV=1
CHS	Sb05g020200.1	0.02	0.00	20.34	Chalcone synthase 1 OS=Sorghum bicolor GN=CHS1 PE=3 SV=1
CHS	Sb05g020160.1	0.09	0.06	23.45	Chalcone synthase 4 OS=Sorghum bicolor GN=CHS4 PE=3 SV=1
CHS	Sb05g020210.1	0.02	0.00	13.86	Chalcone synthase 3 OS=Sorghum bicolor GN=CHS3 PE=3 SV=1
CHS	Sb05g020220.1	0.02	0.04	9.17	Chalcone synthase 7 OS=Sorghum bicolor GN=CHS7 PE=3 SV=1
CHS	Sb05g020150.1	6.86	7.64	58.56	Chalcone synthase 5 OS=Sorghum bicolor GN=CHS5 PE=3 SV=1
CHS	Sb07g004700.1	0.00	0.00	20.33	Chalcone synthase 1 OS=Oryza sativa subsp. japonica GN=CHS1 PE=2 SV=1
CHI	Sb08g002620.2	1.57	9.48	134.42	chalcone-flavanone isomerase-like proetin [Arabidopsis thaliana]
CHI	Sb08g002620.1	1.22	7.04	103.65	chalcone-flavanone isomerase-like proetin [Arabidopsis thaliana]
FNSII	Sb02g000220.1	1.38	0.83	40.47	Cytochrome P450 93A3 OS=Glycine max GN=CYP93A3 PE=2 SV=1
F3'H	Sb04g024710.1	0.12	0.50	28.21	Flavonoid 3'-monooxygenase OS=Petunia hybrida GN=CYP75B2 PE=2 SV=1
F3'H	Sb04g024730.1	0.48	0.87	5.30	Flavonoid 3'-monooxygenase OS=Petunia hybrida GN=CYP75B2 PE=2 SV=1
F3'H	Sb04g024750.1	0.45	0.32	1.82	Flavonoid 3'-monooxygenase OS=Arabidopsis thaliana GN=CYP75B1 PE=1 SV=1
ANR	Sb06g029550.1	0.10	0.08	80.14	Anthocyanidin reductase OS=Arabidopsis thaliana GN=BAN PE=1 SV=2
Methyl transferase	Sb10g027360.1	0.02	0.12	77.05	5-pentadecatrienyl resorcinol O-methyltransferase OS=Sorghum bicolor GN=OMT3 PE=1 SV=1
Methyl transferase	Sb07g004690.1	0.06	0.20	153.96	Eugenol O-methyltransferase OS=Sorghum bicolor GN=EOMT PE=1 SV=1
Methyl transferase	Sb10g027340.1	0.00	0.00	29.73	5-pentadecatrienyl resorcinol O-methyltransferase OS=Sorghum bicolor GN=OMT3 PE=1 SV=1
Methyl transferase	Sb05g026710.1	0.06	0.11	17.33	O-methyltransferase ZRP4 OS=Zea mays GN=ZRP4 PE=2 SV=1
Methyl transferase	Sb07g004710.1	0.02	0.03	30.28	Eugenol O-methyltransferase OS=Sorghum bicolor GN=EOMT PE=1 SV=1
Methyl transferase	CUFF.2703.1	0.00	0.02	27.35	5-pentadecatrienyl resorcinol O-methyltransferase OS=Sorghum bicolor GN=OMT3 PE=1 SV=1
Methyl transferase	Sb01g042580.1	0.65	1.82	3.05	Homocysteine S-methyltransferase 1 OS=Zea mays GN=HMT-1 PE=2 SV=1
Methyl transferase	Sb03g012610.1	0.07	0.03	1.17	S-adenosyl-L-methionine-dependent methyltransferase-like protein [Arabidopsis thaliana]
Methyl transferase	Sb03g032930.1	15.69	10.77	34.09	S-adenosyl-L-methionine-dependent methyltransferase domain-containing protein [Arabidopsis thaliana]
Methyl transferase	Sb04g003110.1	152.55	236.35	347.61	Benzoate carboxyl methyltransferase OS=Antirrhinum majus GN=BAMT PE=1 SV=1
Methyl transferase	Sb01g034010.1	18.94	22.50	28.05	Probable methyltransferase PMT2 OS=Arabidopsis thaliana GN=At1g26850 PE=1 SV=2
CCR	Sb07g021680.1	32.99	33.70	53.07	cinnamoyl coa reductase 1 [Arabidopsis thaliana]
CAD	Sb06g001430.1	0.23	0.33	1.58	Probable cinnamyl alcohol dehydrogenase 6 OS=Oryza sativa subsp. japonica GN=CAD6 PE=2 SV=2
Dirigent	Sb05g008780.1	0.61	0.37	51.98	disease resistance-responsive, dirigent domain-containing protein [Arabidopsis thaliana]
Dirigent	Sb05g008770.1	0.00	0.00	12.83	Disease resistance-responsive (dirigent-like protein) family protein [Arabidopsis thaliana]
Dirigent	Sb05g008800.1	0.09	0.00	26.54	disease resistance-responsive, dirigent domain-containing protein [Arabidopsis thaliana]
Dirigent	Sb05g008790.1	0.39	0.10	26.77	Disease resistance-responsive (dirigent-like protein) family protein [Arabidopsis thaliana]
Dirigent	Sb09g001860.1	0.61	0.74	9.70	-
Dirigent	Sb03g041610.1	5.12	2.04	25.43	disease resistance-responsive, dirigent domain-containing protein [Arabidopsis thaliana]
