# Supplementary material for: Simultaneous Transcriptome Analysis of Sorghum and Bipolaris sorghicola by Using RNA-seq in Combination with De Novo Transcriptome Assembly
Source: PLoS One. 2013 Apr 30;8(4):e62460. doi: 10.1371/journal.pone.0062460 (PMC3640049; doi:10.1371/journal.pone.0062460)
Supplement: Table S5 — Pathogen ( Bipolaris sorghicola )-induced genes in Sorghum bicolor encoding pathogenesis-related (PR) proteins. (PDF) [file pone.0062460.s006.pdf]

Table S5
Pathogen (*Bipolaris sorghicola*)-induced genes in *Sorghum bicolor* encoding pathogenesis-related (PR) proteins

Family	Transcript	RPKM<br>Control	 Mock	 Infected	Annotation
PR-1 like	Sb02g002150.1	0.08	0.04	314.65	Pathogenesis-related protein PRB1-2 OS=Hordeum vulgare PE=2 SV=1
PR-1 like	Sb10g001940.1	36.97	52.98	152.78	Pathogenesis-related protein PRMS OS=Zea mays GN=PRMS PE=2 SV=1
β-1, 3-Glucanase	Sb03g045460.1	1.32	4.25	336.68	Glucan endo-1,3-beta-glucosidase, acidic isoform OS=Zea mays PE=2 SV=1
β-1, 3-Glucanase	Sb08g019670.1	6.88	9.20	129.66	Glucan endo-1,3-beta-glucosidase GII OS=Hordeum vulgare PE=1 SV=1
β-1, 3-Glucanase	Sb03g045450.1	1.02	0.85	5.73	Glucan endo-1,3-beta-glucosidase, acidic isoform OS=Zea mays PE=2 SV=1
β-1, 3-Glucanase	Sb03g045490.1	125.57	118.02	150.40	Glucan endo-1,3-beta-glucosidase GII OS=Hordeum vulgare PE=1 SV=1
Chitinase	Sb06g021250.1	7.15	13.44	173.82	Endochitinase A OS=Zea mays PE=1 SV=1
Chitinase	Sb06g021240.1	3.34	6.36	33.57	Endochitinase A OS=Zea mays PE=1 SV=1
Chitinase	Sb06g021260.1	0.00	0.21	6.41	Endochitinase A OS=Zea mays PE=1 SV=1
Chitinase	Sb04g025430.1	1.55	2.52	17.10	Chitinase 6 OS=Oryza sativa subsp. japonica GN=Cht6 PE=2 SV=1
Chitinase	Sb3219s002010.1	288.84	171.04	431.79	Chitinase 12 OS=Oryza sativa subsp. japonica GN=Cht12 PE=2 SV=2
Chitinase	Sb09g019660.1	14.04	8.58	43.63	Chitinase 2 OS=Oryza sativa subsp. japonica GN=Cht2 PE=1 SV=1
Chitinase	Sb03g030100.1	23.76	21.58	551.50	Acidic endochitinase OS=Vitis vinifera GN=CHIT3 PE=2 SV=1
Chitinase	Sb01g021920.1	3.18	1.85	36.92	Chitinase 2 OS=Tulipa bakeri PE=1 SV=1
Chitinase	Sb05g006880.1	38.08	26.01	330.72	Chitinase 1 OS=Tulipa bakeri PE=1 SV=1
Barwin	Sb05g022940.1	2.54	2.27	654.76	Barwin OS=Hordeum vulgare PE=1 SV=1
Barwin	Sb05g022960.1	64.75	70.74	171.28	Barwin OS=Hordeum vulgare PE=1 SV=1
Thaumatin	Sb08g022440.1	1.06	0.49	1092.41	Thaumatin-like protein OS=Oryza sativa subsp. japonica GN=Os12g0628600 PE=1 SV=1
Thaumatin	Sb08g022420.1	0.14	0.11	306.90	Thaumatin-like protein OS=Oryza sativa subsp. japonica GN=Os12g0628600 PE=1 SV=1
Thaumatin	Sb01g012710.1	2.19	1.13	375.80	Zeamatin OS=Zea mays GN=Zlp PE=1 SV=2
Thaumatin	Sb08g022430.1	0.43	0.14	220.84	Thaumatin-like pathogenesis-related protein 4 OS=Avena sativa GN=RASTL-4 PE=2 SV=1
Thaumatin	Sb08g022450.1	2.08	4.01	216.61	Thaumatin-like protein OS=Oryza sativa subsp. japonica GN=Os12g0628600 PE=1 SV=1
Thaumatin	Sb01g012700.1	0.09	0.16	27.09	Protein P21 OS=Glycine max PE=1 SV=1
Thaumatin	Sb08g022400.1	0.26	0.61	29.36	Thaumatin-like protein OS=Oryza sativa subsp. japonica GN=Os12g0628600 PE=1 SV=1
Thaumatin	Sb08g022390.1	2.35	2.04	45.07	Thaumatin-like pathogenesis-related protein 4 OS=Avena sativa GN=RASTL-4 PE=2 SV=1
Thaumatin	Sb08g022490.1	0.00	0.00	23.92	Thaumatin-like protein OS=Oryza sativa subsp. japonica GN=Os12g0628600 PE=1 SV=1
Thaumatin	Sb02g037350.1	27.98	44.28	165.83	Zeamatin OS=Zea mays GN=Zlp PE=1 SV=2
Thaumatin	Sb08g022410.1	42.74	50.95	59.86	Thaumatin-like protein OS=Oryza sativa subsp. japonica GN=Os12g0628600 PE=1 SV=1
Bet v I allergen	Sb01g037970.1	1.98	2.24	689.78	Pathogenesis-related protein 1 OS=Asparagus officinalis GN=PR1 PE=2 SV=1
Bet v I allergen	Sb01g037920.2	0.08	0.22	186.11	Pathogenesis-related protein 1 OS=Asparagus officinalis GN=PR1 PE=2 SV=1
Bet v I allergen	Sb01g037940.1	0.12	0.16	136.58	Pathogenesis-related protein 1 OS=Asparagus officinalis GN=PR1 PE=2 SV=1
Bet v I allergen	Sb01g037920.1	0.06	0.45	126.68	Pathogenesis-related protein 1 OS=Asparagus officinalis GN=PR1 PE=2 SV=1
Bet v I allergen	Sb01g037960.1	2.11	0.77	234.21	Pathogenesis-related protein 1 OS=Asparagus officinalis GN=PR1 PE=2 SV=1
Bet v I allergen	CUFF.3050.2	0.15	0.11	65.60	Pathogenesis-related protein 1 OS=Asparagus officinalis GN=PR1 PE=2 SV=1
Bet v I allergen	Sb01g037950.1	1.92	0.96	80.99	Pathogenesis-related protein 1 OS=Asparagus officinalis GN=PR1 PE=2 SV=1
